# Supplementary material for: Unveiling the Immunogenicity of Ovarian Tumors as the Crucial Catalyst for Therapeutic Success
Source: Cancers (Basel). 2023 Dec 2;15(23):5694. doi: 10.3390/cancers15235694 (PMC10705691; doi:10.3390/cancers15235694)
Supplement: Supplementary file 1 [file cancers-15-05694-s001.zip › cancers-2727879-supplementary.pdf]

Supplementary Table S1: Current clinical trials addressing some components affecting EOC immunogenicity

Clinical trial ID	Phase	Line	Treatment	Diseases treated	References
**NCT01112293**	II	Relapsed	Anti-TGF Monoclonal Antibody (GC1008)	Pleural Malignant Mesothelioma	[222]
**NCT04935359**	III	First line	NIS793 in Combination with Gemcitabine and Nab-paclitaxel	Metastatic PDAC	[223]
**NCT05145569**	I	Metastatic	Neoadjuvant Chemotherapy (Carboplatin AUC 5 and paclitaxel) With or Without BINTRAFUSP ALFA (M7824)	Metastatic Advanced OC	NA
**NCT02538471**	II	Metastatic	LY2157299 Monohydrate (LY2157299) and Radiotherapy	Metastatic Breast Cancer	[224]
**NCT04429542**	I	First line	Bifunctional EGFR/TGFβ Fusion Protein BCA101 Monotherapy and in Combination Therapy	EGFR-Driven Advanced Solid Tumors (HNSCC, SCCL, cSCC, SCC EOC, PC)	[200,225]
**NCT00063401**	II	First line	Cetuximab (C225)/Paclitaxel/Carboplatin	Advanced Stage Ovarian, Primary Peritoneal, and Fallopian Tube Cancer	NA
**NCT02954523**	I/II	First line	Dasatinib and Osimertinib (AZD9291)	Advanced NSCLC With EGFR Mutations	[201]
**NCT04511871**	I	Relapsed/ refractory	CAR modified autologous T cells (CCT303-406)	HER2 Positive Solid Tumors	Study still enrolling
**NCT04562298**	I	Relapsed/ refractory	CAR-T cell therapy (LCAR-M23) targeting MSLN	EOC	NA
**NCT02580747**	I	Relapsed/ Chemo refractory	Patient autologous or donor-derived T cells transduced with the anti-meso vector (CART-meso cells)	Advanced Malignancies: malignant mesothelioma, OC, PC, TNBC, EC, and other mesothelin positive tumors	NA
**NCT01583686**	I/II	Metastatic	Lymphodepleting conditioning followed by infusion of anti-mesothelin gene engineered T lymphocytes	Metastatic or unresectable cancer expressing mesothelin	NA
**NCT05211557**	I	Relapsed	Fully Human B7H3 (CD276) CAR-T cells	Recurrent Malignant OC	Study still enrolling
**NCT05472857**	I	Advanced	CLDN18.2-targeting CAR T Cells	Solid tumors with positive CLDN18.2 expression (GC, PC, OC, gastroesophageal junction adenocarcinoma)	Study still enrolling
**NCT05225363**	I	Platinum resistant	TAG72-CAR T Cells	Platinum Resistant EOC	Study still enrolling

**NCT05613088**	II	Platinum resistant	Farletuzumab ecteribulin (MORAb-202, Folate Receptor Alpha-targeting Antibody-drug Conjugate) compared to investigator choice of chemotherapy	HGSC, primary peritoneal, or fallopian tube cancer	Study still enrolling
**NCT04300556**	I/II	Platinum resistant	Farletuzumab Ecteribulin	OC, endometrial cancer, NSCLC, TNBC	Study still enrolling
**NCT02671435**	I/II	Metastatic	Durvalumab (MEDI4736) and Monalizumab	Advanced solid tumors	NA
**NCT05769959**	I/II	Metastatic	Study of RO7515629	Advanced or metastatic solid tumors expressing HLA-G (RCC, NSCLC, pancreatic adenocarcinoma, CC, ovarian neoplasms)	Study still enrolling
**NCT00529022**	I	Platinum resistant	Azacitidine and Valproic Acid Plus Carboplatin	Platinum Resistant EOC	NA
**NCT02900560**	II	Platinum resistant/ refractory	Pembrolizumab With or Without oral azacitidine (CC-486)	EOC	NA

Abbreviations: Pancreatic ductal adenocarcinoma [PDAC], ovarian cancer [OC], epithelial ovarian cancer [EOC], head and neck squamous cell carcinoma [HNSCC], squamous cell carcinoma of the lung [SCCL], cutaneous squamous cell carcinoma [cSCC], squamous cell carcinoma [SCC], pancreatic cancer [PC], endometrial cancer [EC], high-grade serous ovarian cancer [HGSC], non-small cell lung carcinoma [NSCLC], gastric cancer [GC], triple-negative breast cancer [TNBC]), renal adenocarcinoma [RCC], colorectal cancer [CC], NA: No publications available.

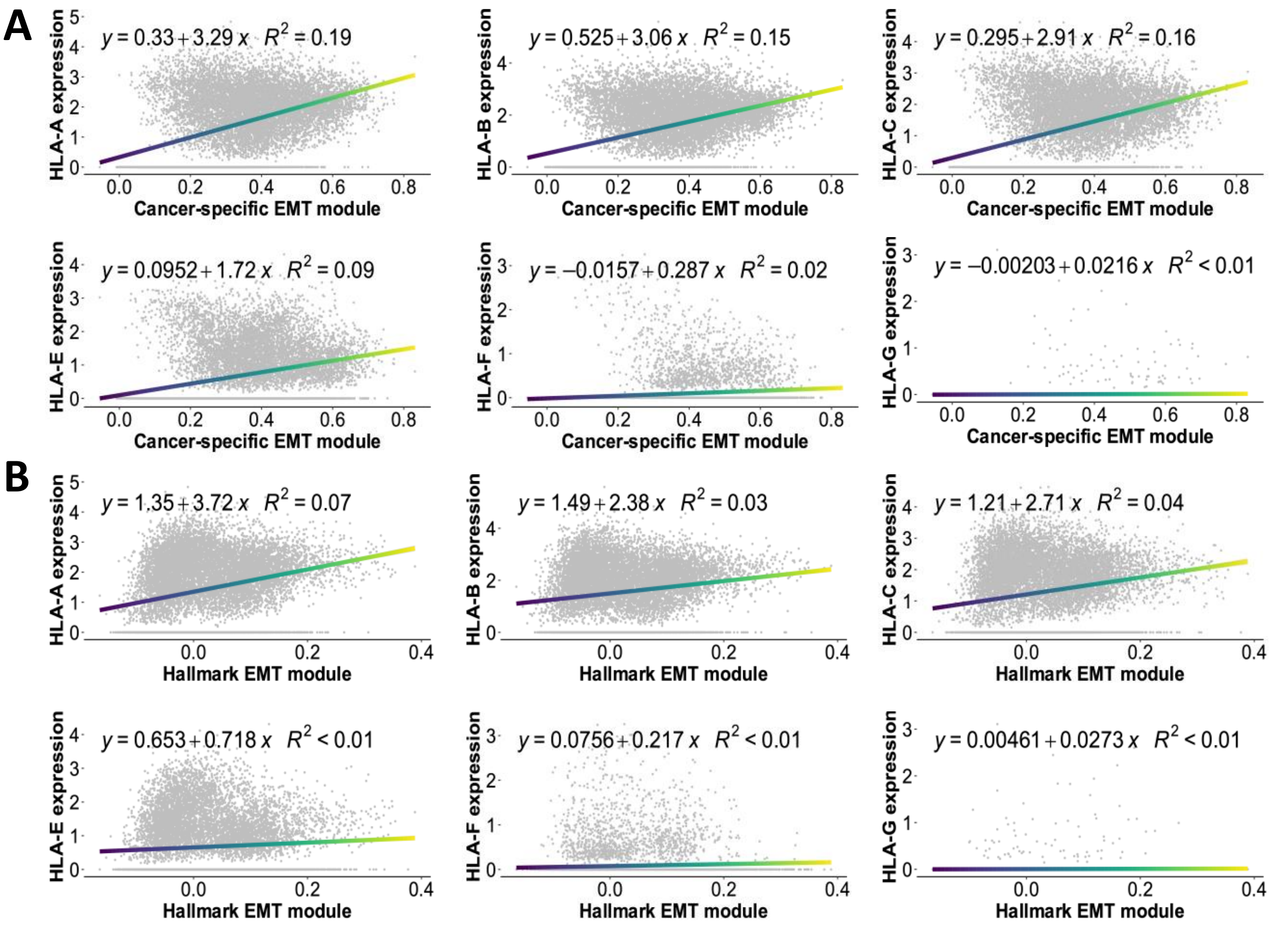

Supplementary Figure S1. Average gene expression of HLA I allotypes increases in correlation with the average EMT gene expression in ovarian cancer cells. Gene expression of HLA I allotypes in ovarian cancer cells.
